# Use of estimated glomerular filtration rate to predict incident chronic kidney disease in patients at risk of cardiovascular disease: a retrospective study

**DOI:** 10.1186/s12882-019-1494-8

**Published:** 2019-08-20

**Authors:** Saif Al-Shamsi, Abderrahim Oulhaj, Dybesh Regmi, Romona D. Govender

**Affiliations:** 1 Department of Internal Medicine, College of Medicine and Health Sciences, United Arab Emirates University, Al Ain, United Arab Emirates; 2 Institute of Public Health, College of Medicine and Health Sciences, United Arab Emirates University, Al Ain, United Arab Emirates; 3 Department of Family Medicine, College of Medicine and Health Sciences, United Arab Emirates University, Al Ain, United Arab Emirates

**Keywords:** Chronic kidney disease, Estimated glomerular filtration rate, Cardiovascular disease, Prediction, Nomogram, Sub-distribution hazards model

## Abstract

**Background:**

Patients with cardiovascular disease are at an increased risk of chronic kidney disease (CKD). However, data on incident CKD in patients with multiple vascular comorbidities are insufficient. In this study, we identified the predictors of CKD stages 3–5 in patients at risk of cardiovascular disease and used their estimated glomerular filtration rate (eGFR) to construct a nomogram to predict the 5-year risk of incident CKD.

**Methods:**

Ambulatory data on 622 adults with preserved kidney function and one or more cardiovascular disease risk factors who attended outpatient clinics at a tertiary care hospital in Al-Ain, United Arab Emirates were obtained retrospectively. eGFR was calculated using the Chronic Kidney Disease Epidemiology Collaboration equation and assessed every 3 months from baseline to December 12, 2017. Fine and Gray competing risk regression model was used to identify the independent variables and construct a nomogram to predict incident CKD at 5 years, which is defined as eGFR < 60 mL/min/1.73 m^2^ for ≥3 months. Time-dependent area under the receiver operating characteristic curve (AUC) was used to evaluate the discrimination ability of the model. Calibration curves were applied to determine the calibration ability and adjusted for the competing risk of death. Internal validation of predictive accuracy was performed using K-fold cross-validation.

**Results:**

Of the 622 patients, 71 had newly developed CKD stages 3–5 over a median follow-up of 96 months (interquartile range, 86–103 months). Baseline eGFR, hemoglobin A1c, total cholesterol, and history of diabetes mellitus were identified as significant predictors of CKD stages 3–5. The nomogram had good discrimination in predicting the disease stages, with a time-dependent AUC of 0.918 (95% confidence interval, 0.846–0.964) at 5 years, after internal validation by cross-validation.

**Conclusions:**

This study demonstrated that incident CKD could be predicted with a simple and practical nomogram in patients at risk of cardiovascular disease and with preserved kidney function, which in turn could help clinicians make more informed decisions for CKD management in these patients.

**Electronic supplementary material:**

The online version of this article (10.1186/s12882-019-1494-8) contains supplementary material, which is available to authorized users.

## Background

Cardiovascular disease (CVD) is the leading cause of death worldwide [1] and an important risk factor for chronic kidney disease (CKD) [2]. A diminished estimated glomerular filtration rate (eGFR) has been shown to increase the risk of CVD morbidity and mortality [3]. Approximately 1 in 10 people worldwide have CKD [4]. The marked increase in CKD prevalence over the past two decades could be explained by the rising incidence of chronic non-communicable diseases, such as diabetes mellitus (DM), hypertension (HTN), obesity, and dyslipidemia [5, 6]. Much attention has been focused on the significant observation that CVD risk and mortality in patients with CKD is increased [7]. CVD and CKD share numerous risk factors, which suggests that patients with CVD also have an increased risk of CKD. CVD may promote the initiation and progression of CKD, for example, through decreased renal perfusion due to atherosclerosis of the renal arteries [2]. The National Kidney Foundation Kidney Disease Outcomes Quality Initiative guidelines recommend that the eGFR be calculated in patients at risk for early detection of CKD and to prevent disease progression [8].

However, despite the increasing CVD prevalence, data on CKD progression in patients at risk of CVD are limited [3, 9]. Therefore, we aimed to assess the risk of developing CKD stages 3–5 using baseline eGFR in patients with vascular comorbidities and to develop a nomogram to predict the 5-year risk of incident CKD for clinical use.

## Methods

### Patients and procedures

This is a retrospective cohort study of United Arab Emirates (UAE) nationals who visited the outpatient clinics at Tawam Hospital between April 1, 2008, and December 31, 2008, and had either CVD or one or more CVD risk factors. Tawam Hospital is a state-funded tertiary care facility located in Al Ain, UAE. This medical center and its outpatient clinics serve a population of approximately 770,000, the majority of whom are UAE nationals [10]. The study protocol was approved by Tawam Hospital and the United Arab Emirates University research and ethics board (IRR536/17). The requirement for informed consent was waived because patient records and information were anonymized and de-identified prior to analysis.

Sociodemographic data and clinical information were manually extracted from 1118 patients’ ambulatory electronic medical records (EMRs) that were available for review. Inclusion criteria for this study were UAE nationals ≥18 years old with any of the following conditions at baseline: HTN, CVD, DM, dyslipidemia, history of smoking, body mass index (BMI) ≥25 kg/m^2^, systolic blood pressure (SBP) ≥120 mmHg, diastolic blood pressure (DBP) ≥80 mmHg, serum glycosylated hemoglobin A1c (HbA1c) ≥5.7%, serum triglycerides (TG) ≥2.26 mmol/L, or serum total cholesterol (TC) ≥6.21 mmol/L.

**Fig. 1:**
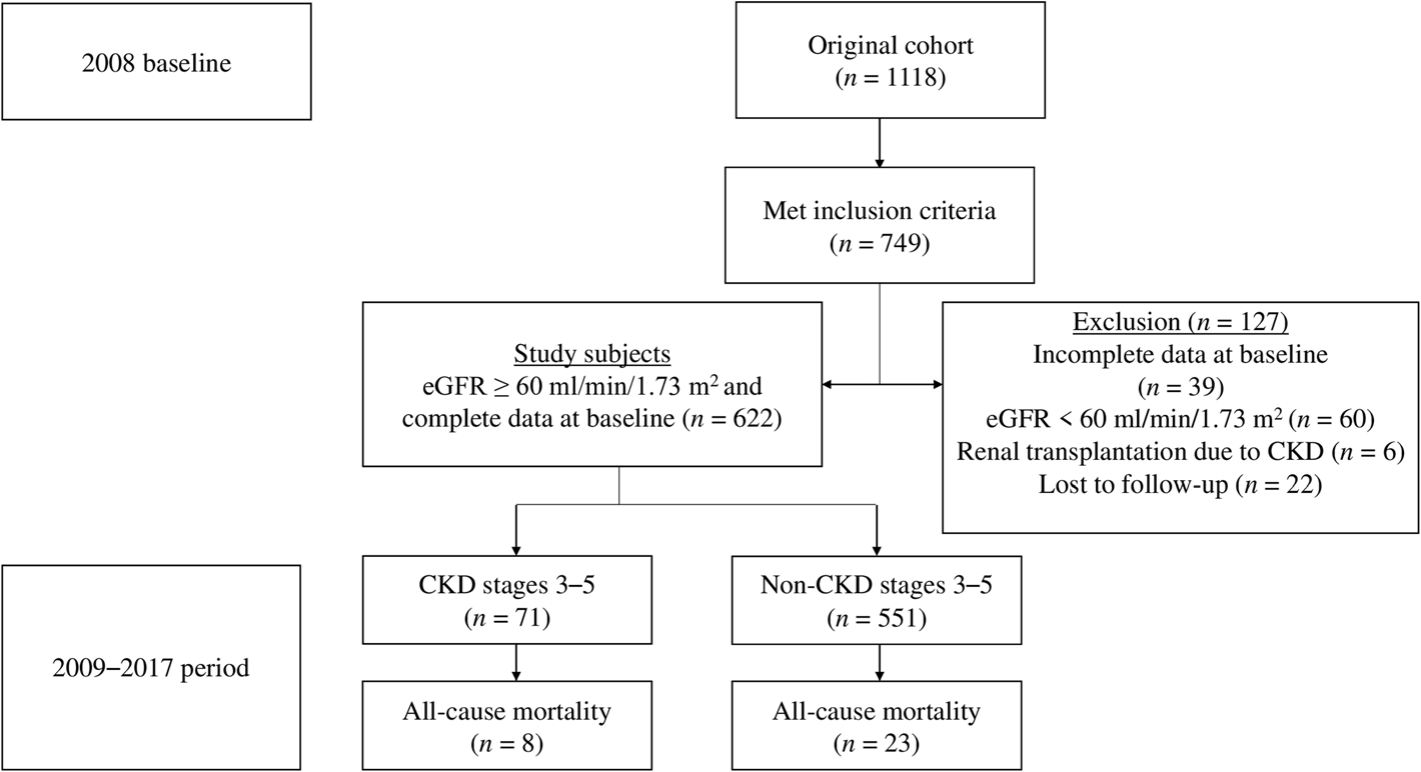
Flow diagram of the patient population. *CKD* chronic kidney disease, *eGFR* estimated glomerular filtration rate

Of the 749 eligible patients who met the inclusion criteria, 105 were excluded (60 had eGFR < 60 mL/min/1.73 m^2^, 6 were renal transplant recipients, and 39 had missing data on baseline serum creatinine (SCr), TG, or HbA1c levels) (Fig. 1). The eGFR was repeatedly assessed for each patient every 3 months from baseline to December 12, 2017. Twenty-two patients had not undergone a repeat SCr measurement during the follow-up period; they were considered to have been lost to follow-up and thus were excluded from the final analysis. A total of 622 patients with eGFR ≥60 mL/min/1.73 m^2^ were finally enrolled in this study.

### Definitions

BMI was calculated as weight (kg) divided by height (m^2^). Obesity was defined as BMI ≥30 kg/m^2^. Patients receiving antihypertensive medications were considered as having HTN. Similarly, patients with dyslipidemia were those taking lipid-lowering medications, and patients with DM were those receiving antidiabetic medications. Smoking history was positive if there was a current or any history of smoking tobacco. Patients were considered to have vascular disease if they had a diagnosis of coronary heart disease (angina, prior myocardial infarction, angioplasty of the coronary arteries, or coronary artery surgery), cerebrovascular accident, or peripheral arterial disease. All-cause mortality was defined as death from any cause.

### Outcomes

In this study, CKD stages 3–5 were defined as eGFR < 60 mL/min/1.73 m^2^ for ≥3 months [8]. eGFR was calculated using the CKD Epidemiology Collaboration (CKD-EPI) creatinine equation [11]. All deaths were confirmed through review of hospital records and death certificate data. Non-CKD death, including death from CVD, cancers, and other causes, was classified as competing events.

Baseline and follow-up laboratory tests were performed at Tawam Hospital’s Medical Laboratory Department. The Synchron Clinical System (UniCel DxC-800; Beckman Coulter, Inc., Fullerton, CA) was used to measure fasting lipid profile and SCr level by standard methods, while HbA1c levels were assessed using the automated analyzer Integra 400 Plus (Roche Diagnostics, Mannheim, Germany). The recommended manufacturer’s reference intervals for SCr level were 53–115 μmol/L and 58–96 μmol/L for men and women, respectively.

### Statistical analyses

The baseline clinical variables and demographic data recorded in the patients’ EMRs and retrieved for analysis were age; sex; history of DM, HTN, dyslipidemia, smoking, and CVD; SBP; DBP; BMI; TC; TG; HbA1c; and eGFR. The baseline characteristics of patients who did and did not develop CKD stages 3–5 were compared using the independent samples t-test for normally distributed continuous variables, the Mann-Whitney U test for non-normally distributed continuous variables, and Fisher’s exact test (two-tailed) for categorical variables. We did not use any method of data imputation, and missing covariate and dependent variable data were excluded from the analysis.

Time of follow-up for each patient was calculated from the baseline visit in 2008 to either incident CKD, death, or the last outpatient clinic visit, whichever occurred first. Considering the potential bias due to the competing risk of non-CKD death, we used the Fine and Gray regression model to adjust for the risk estimates of non-CKD death as a competing risk [12]. The unadjusted cumulative incidence function in the presence of competing risk events was examined to compare the probability of failure over the follow-up period across eGFR categories (i.e., 60–89 mL/min/1.73 m^2^, 90–99 mL/min/1.73 m^2^, and ≥ 100 mL/min/1.73 m^2^).

Using the cmprsk package in R software [13], we constructed the prediction model with the following steps. First, univariate Fine and Gray regression models were used on all 14 candidate variables. Variables with a statistical significance of the estimated regression coefficients of *P* > 0.2 were removed. Second, all significant variables were included to develop the multivariate model. Third, we constructed two CKD risk prediction models: one based on all remaining variables and the other using backward-stepwise selection. For each model, sub-distribution hazard ratios (SHRs) and 95% confidence intervals (95% CIs) were calculated to estimate the relative risk. The proportional hazards assumption was assessed by examining plots of the scaled Schoenfeld residuals against time failure for the predictors. Multicollinearity was evaluated by examining tolerance.

Moreover, two important components of predictive accuracy, i.e., discrimination and calibration, were used to evaluate the performance of the models [14]. The evaluation was performed using the package survival [15] and package ggplot2 in R [16]. Time-dependent area under the receiver operating characteristic curve (AUC) was employed to assess and compare the discriminative ability of the two models [17, 18]. Larger AUC values indicate better overall performance. An AUC of 0.5 indicates no predictive ability, whereas a value of 1 represents perfect predictive ability. The calibration plot, which is obtained using cross-validation method, was used to compare the predicted probability with the observed probability in both models. Internal validation of predictive accuracy was performed by applying a cross-validation approach using 1000 splits of the data into training and validation set. The prediction model with good performance was implemented into a nomogram using the mstate package and rms package in R [19, 20].

All statistical analyses and data manipulations were performed using R software version 3.5.2 (The R Foundation, Vienna, Austria) and IBM®SPSS® software, version 25 (IBM Corporation, Armonk, NY, USA). All *P* values were two-tailed, and *P* values < 0.05 were considered statistically significant.

## Results

### Baseline characteristics and follow-up

Table 1 presents the baseline characteristics of the cohort and compares these characteristics according to the development of CKD stages 3–5 event. In our cohort of 622 patients, 71 (11.4%) had newly developed CKD stages 3–5 over a median follow-up (interquartile range) of 96 months (86–103 months). During the study period, 31 (5.0%) patients died (Fig. 1). The annual all-cause mortality rate over the study period was 6.2 per 1000 individuals (95% CI, 4.4–8.7) per year. The mean age in this study population was 52.38 ± 14.48 years, and half were men. Approximately 60% of patients had hypertension, and almost half of the cohort had obesity. Around one third had DM and 14% had a history of CVD at baseline. The mean eGFR of the cohort was 98.99 ± 19.36 mL/min/1.73 m^2^. Moreover, patients with CKD stages 3–5 event were older at baseline; more frequently had a history of CVD, DM, HTN, and dyslipidemia; and had a higher SBP, TG, and HbA1c, but had a lower DBP, TC, and eGFR, than patients without CKD stages 3–5 event.

**Table 1:** Comparison of baseline characteristics according to the development of CKD stages 3–5

Characteristic	Total (*n* = 622)	CKD^a^ (*n* = 71)	No CKD^a^ (*n* = 551)	*P* value^b^
Age (years)	52.38 ± 14.48	63.35 ± 9.49	50.96 ± 14.41	< 0.001
Male sex, *n* (%)	312 (50.2)	41 (57.7)	271 (49.2)	0.207
History of, *n* (%)				
CVD	87 (14.0)	26 (36.6)	61 (11.1)	< 0.001
Smoking	92 (14.8)	15 (21.1)	77 (14.0)	0.112
Obesity	294 (47.3)	38 (53.5)	256 (46.5)	0.312
DM	197 (31.7)	48 (67.6)	149 (27.0)	< 0.001
HTN	368 (59.2)	60 (84.5)	308 (55.9)	< 0.001
Dyslipidemia	318 (51.1)	54 (76.1)	264 (47.9)	< 0.001
Anthropometric values				
BMI (kg/m^2^)	30.40 ± 6.28	30.26 ± 5.95	30.41 ± 6.33	0.847
SBP (mmHg)	131.73 ± 16.46	136.83 ± 18.45	131.07 ± 16.09	0.005
DBP (mmHg)	77.30 ± 11.44	74.55 ± 12.38	77.65 ± 11.28	0.031
Laboratory values				
TC (mmol/L)	5.00 (4.30, 5.80)	4.40 (3.90, 5.45)	5.00 (4.30, 5.80)	0.004
TG (mmol/L)	1.11 (0.79, 1.65)	1.35 (1.01, 1.89)	1.09 (0.78, 1.61)	0.002
SCr (μmol/L)	67.44 ± 17.80	82.29 ± 17.04	65.53 ± 16.99	< 0.001
eGFR (mL/min/1.73 m^2^)	98.99 ± 19.36	78.39 ± 11.58	101.65 ± 18.55	< 0.001
HbA1c (%)	6.10 (5.60, 6.96)	7.30 (6.45, 9.25)	6.00 (5.59, 6.70)	< 0.001

*CVD* cardiovascular disease, *DM* diabetes mellitus, *HTN* hypertension, *BMI* body mass index, *eGFR* estimated glomerular filtration rate, *SBP* systolic blood pressure, *DBP* diastolic blood pressure, *SCr* serum creatinine, *TC* total cholesterol, *TG* triglycerides, *HbA1c* glycosylated hemoglobin A1C

Data are reported as mean ± standard deviation or percent or median (1st, 3rd quartile)

^a^Chronic kidney disease stages 3–5

^b^Independent samples t-test was used to calculate *P* values for continuous variables and Fisher’s exact test (two-tailed) for categorical variables. The Mann-Whitney U-test was used to compare the median values of TC, TG, and HbA1c

**Fig. 2:**
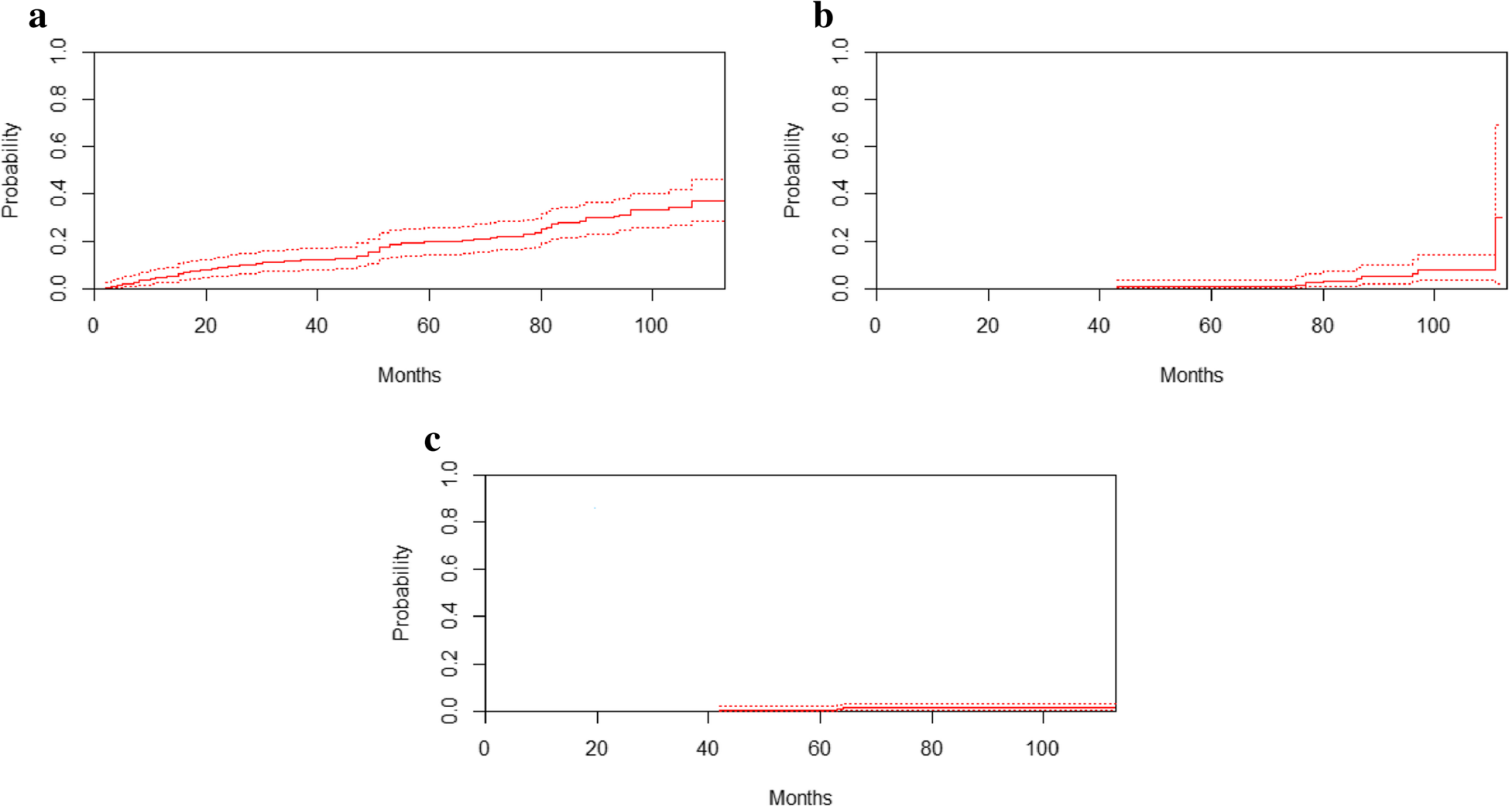
Estimated cumulative incidence curves for CKD stages 3–5. Unadjusted estimated cumulative incidence curves (solid lines) for CKD stages 3–5 in the presence of death as a competing event according to eGFR groups with 95% pointwise CIs (broken lines). **a** eGFR, 60–89 mL/min/1.73 m^2^. **b** eGFR, 90–99 mL/min/1.73 m^2^. **c** eGFR, ≥100 mL/min/1.73 m^2^. *CKD* chronic kidney disease, *eGFR* estimated glomerular filtration rate, *CI* confidence interval

Baseline eGFR was found to be a strong predictor of the development of CKD stages 3–5. After 8 years of follow-up, the unadjusted cumulative probability of developing CKD stages 3–5 in the presence of competing death events was 33.0% (95% CI, 25.9–40.2%), 7.8% (95% CI, 3.5–14.3%), and 1.1% (95% CI, 0.3–3.0%) for patients with a baseline eGFR between 60 and 89 mL/min/1.73 m^2^, between 90 and 99 mL/min/1.73 m^2^, and ≥ 100 mL/min/1.73 m^2^, respectively (Fig. 2).

### CKD risk prediction models

Univariate analyses, adjusted for competing risk events, were used to regress the sub-distribution hazard of incident CKD stages 3–5 on all 14 candidate variables. All variables, except for BMI, that were significant in the univariate analysis (*P* ≤ 0.2) were entered into the multivariate prediction model; four variables were retained after backward-stepwise selection (Table 2). The scatter plots of the scaled Schoenfeld residuals against time failure revealed no substantial deviation from the proportional hazard assumption. In addition, tolerance ranged from 0.50 to 0.83, indicating an absence of multicollinearity. In the multivariate prediction model, after stepwise selection, a greater risk of incident CKD stages 3–5 was associated with history of DM, lower TC, lower eGFR, and increasing HbA1c level.

**Table 2:** Univariate and multivariate Fine and Gray competing risk regression analyses

Characteristics	Univariate analyses			Multivariate analyses (Full model)^a^			Multivariate analyses (Stepwise model)^b^		
	SHR (95% CI)	Coefficient	*P* value	SHR (95% CI)	Coefficient	*P* value	SHR (95% CI)	Coefficient	*P* value
Age (years)	1.06 (1.05–1.08)	0.06	< 0.001	1.01 (0.98–1.03)	0.01	0.680	–	–	–
Sex									
Female	Ref.	Ref.	–	Ref.	Ref.	–	–	–	–
Male	1.48 (0.93–2.36)	0.39	0.099	0.73 (0.39–1.36)	−0.32	0.320	–	–	–
CVD									
No	Ref.	Ref.	–	Ref.	Ref.	–	–	–	–
Yes	3.91 (2.38–6.40)	1.36	< 0.001	0.74 (0.40–1.38)	−0.30	0.340	–	–	–
Smoking									
No	Ref.	Ref.	–	Ref.	Ref.	–	–	–	–
Yes	1.74 (0.99–3.07)	0.55	0.056	1.84 (0.95–3.57)	0.61	0.072	–	–	–
DM									
No	Ref.	Ref.	–	Ref.	Ref.	–	Ref.	Ref.	–
Yes	5.00 (3.05–8.20)	1.61	< 0.001	2.01 (1.03–3.91)	0.70	0.040	2.17 (1.12–4.21)	0.78	0.022
HTN									
No	Ref.	Ref.	–	Ref.	Ref.	–	–	–	–
Yes	3.58 (1.89–6.81)	1.28	< 0.001	1.20 (0.57–2.53)	0.18	0.640	–	–	–
Dyslipidemia									
No	Ref.	Ref.	–	Ref.	Ref.	–	–	–	–
Yes	2.90 (1.68–4.99)	1.06	< 0.001	1.05 (0.54–2.07)	0.05	0.880	–	–	–
BMI (kg/m^2^)	1.00 (0.96–1.03)	−0.004	0.820	Not applicable^c^	–	–	Not applicable^c^	–	–
SBP (mmHg)	1.02 (1.01–1.04)	0.02	0.006	1.02 (1.00–1.04)	0.02	0.096	–	–	–
DBP (mmHg)	0.98 (0.96–1.00)	−0.02	0.069	0.98 (0.96–1.01)	−0.02	0.200	–	–	–
TC (mmol/L)	0.74 (0.59–0.93)	−0.30	0.009	0.73 (0.59–0.91)	−0.31	0.005	0.82 (0.69–0.96)	−0.20	0.015
TG (mmol/L)	1.14 (1.00–1.29)	0.13	0.051	1.15 (0.92–1.45)	0.14	0.230	–	–	–
eGFR (mL/min/1.73 m^2^)	0.92 (0.90–0.93)	−0.09	< 0.001	0.92 (0.90–0.94)	−0.09	< 0.001	0.92 (0.91–0.94)	−0.08	< 0.001
HbA1c (%)	1.38 (1.29–1.48)	0.32	< 0.001	1.18 (1.02–1.36)	0.16	0.027	1.22 (1.08–1.38)	0.20	0.002

*CVD* cardiovascular disease, *DM* diabetes mellitus, *HTN* hypertension, *BMI* body mass index, *eGFR* estimated glomerular filtration rate, *SBP* systolic blood pressure, *DBP* diastolic blood pressure, *SCr* serum creatinine, *TC* total cholesterol, *TG* triglycerides, *HbA1c* glycosylated hemoglobin A1C, *SHR* sub-distribution hazard ratio, *CI* confidence interval

^a^Sub-distribution hazards model, adjusted for all predictors in the final model with all variables included

^b^Sub-distribution hazards model, adjusted for all predictors in the final model selected using backward-stepwise selection

^c^*P* value > 0.2 in the initial univariate analyses and not included in the multivariate analyses

### Calibration, discrimination, and internal validation

**Fig. 3:**
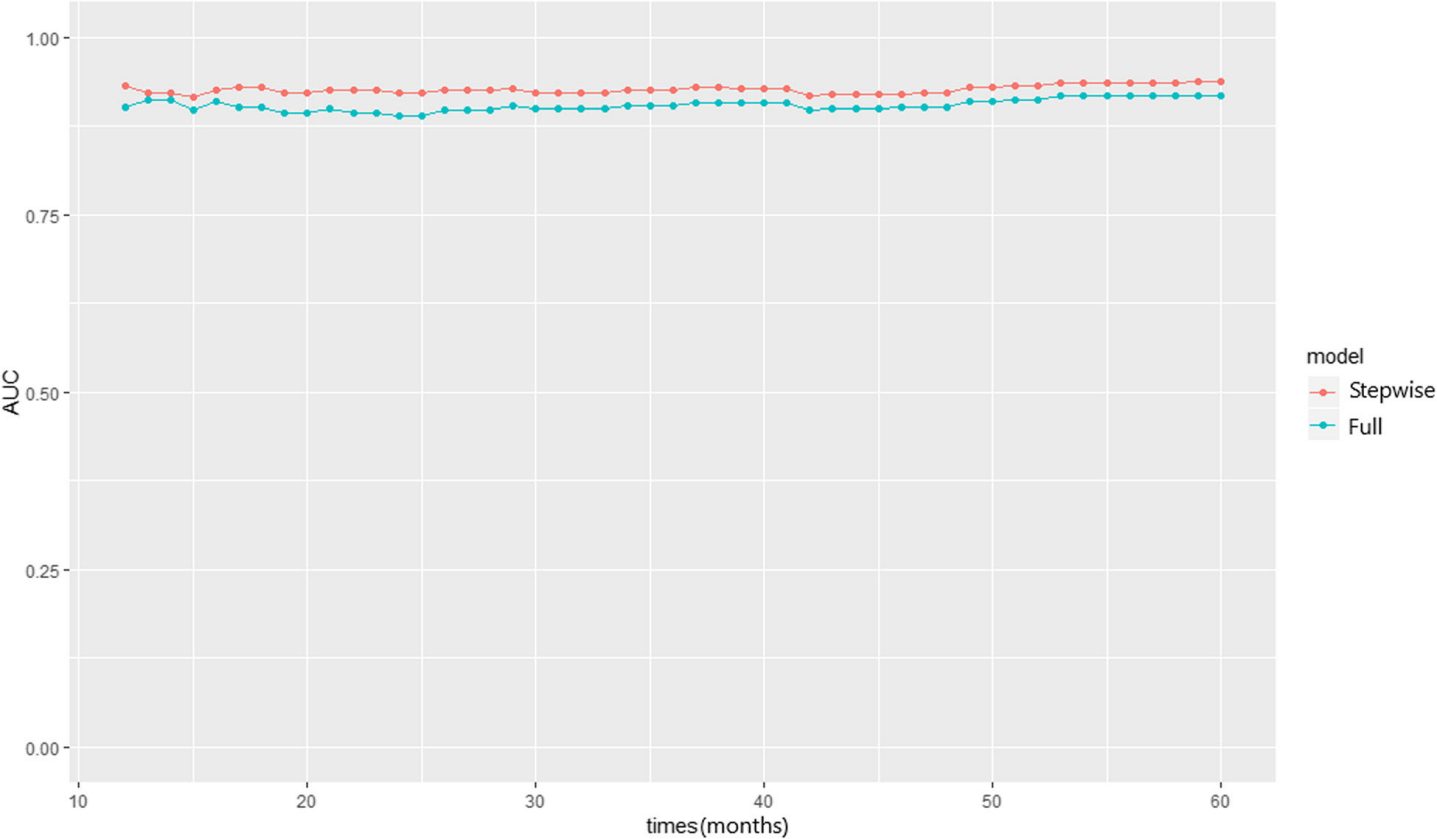
Time-dependent AUC for CKD stages 3–5 risk prediction models *AUC* area under the curve, *CKD* chronic kidney disease

**Fig. 4:**
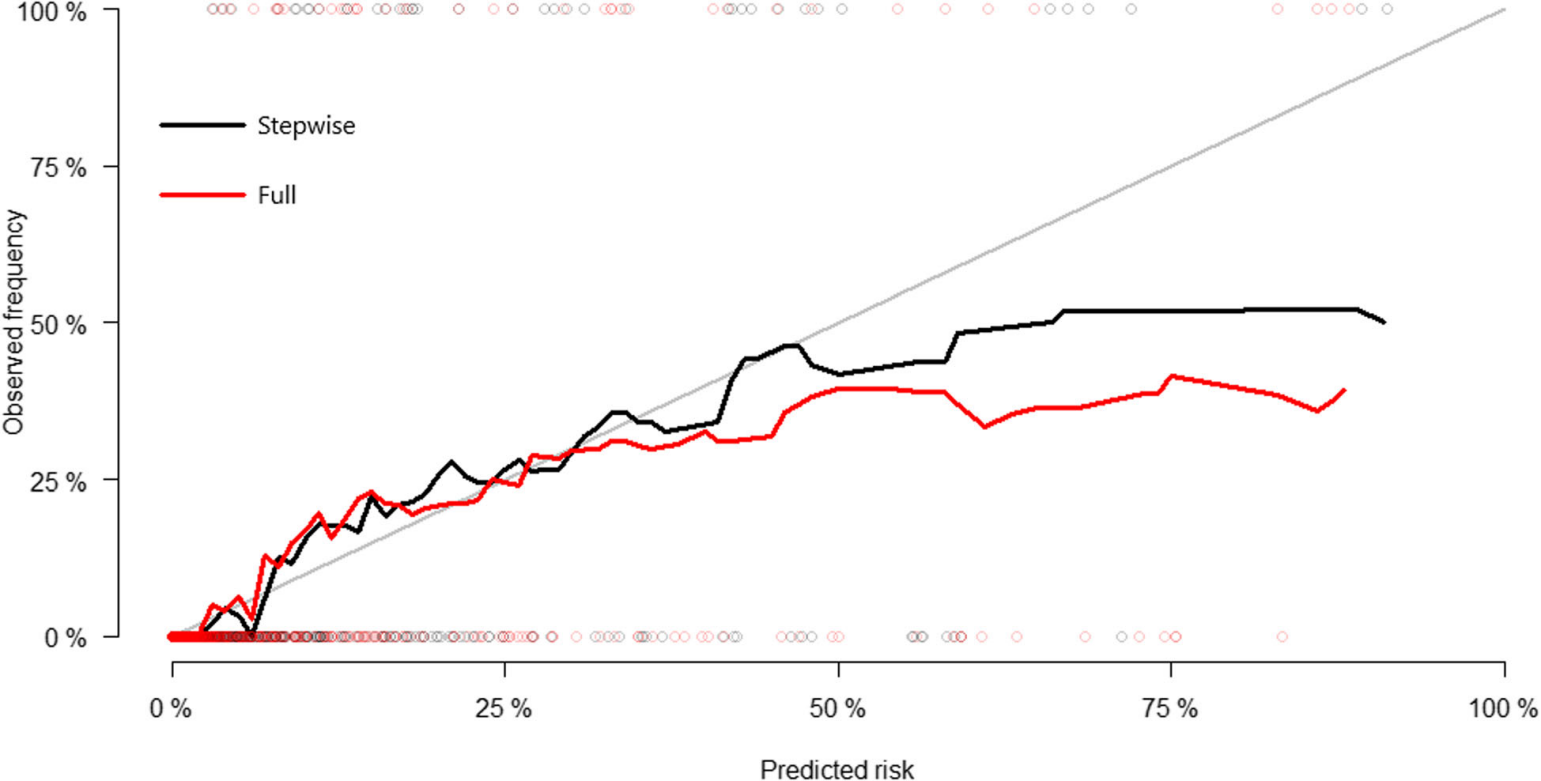
Calibration curves. Fine-Gray regression model after backward-stepwise selection and the full model with all variables included

Following internal validation by cross-validation, the multivariate model after stepwise selection performed better in terms of discrimination and calibration than the multivariate model with all variables included. Time-dependent AUC at 5 years was 0.918 (95% CI, 0.846–0.964) in the multivariate model after stepwise selection and 0.904 (95% CI, 0.853–0.945) in the full multivariate model. Thus, the time-dependent AUC values of the multivariate model after stepwise selection were better than those of the full multivariate model (Fig. 3). The calibration plots comparing actual risk and predicted risk suggested underestimation in the lower risk group and overestimation in the higher risk group in both models (Fig. 4).

### Nomogram

**Fig. 5:**
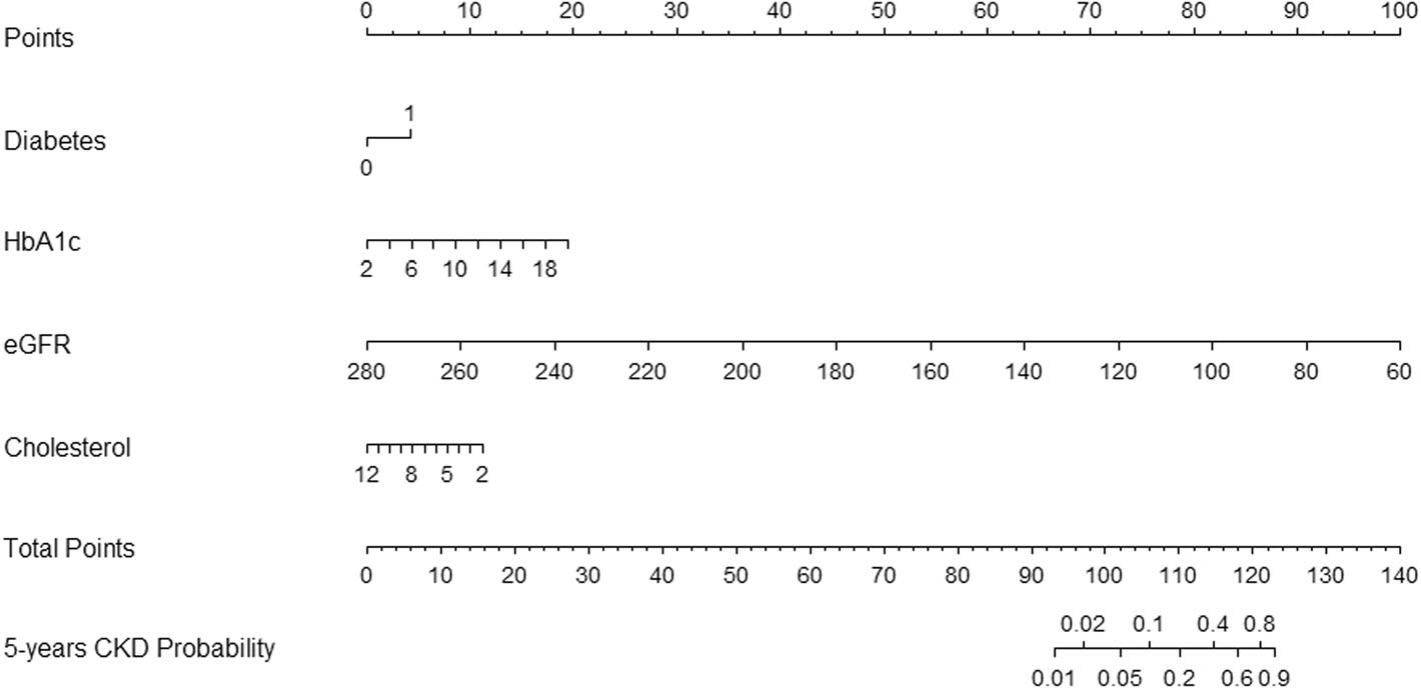
Nomogram to predict the development of CKD stages 3–5 at 5 years *HbA1c* glycosylated hemoglobin A1c, *eGFR* estimated glomerular filtration rate, *CKD* chronic kidney disease

Multivariate Fine and Gray regression model after backward-stepwise selection analysis was selected to build the final prediction model, which identified eGFR, DM, TC, and HbA1c as predictors of incident CKD. A probability nomogram for predicting CKD stages 3–5 within 5 years was constructed using the regression coefficients from the model (Fig. 5). To read the nomogram, a vertical line is drawn up to the points’ axis, and points are assigned for each predictor. The total points are added up, and a vertical line is drawn from the total points’ axis down to the *5-Years CKD Probability* axis, which yields the patient’s overall risk of developing CKD stages 3–5 within 5 years.

The nomogram could be applied in the clinical setting (Additional file 1). For example, a patient with a history of DM, HbA1c of 9%, TC of 3 mmol/L, and eGFR of 65 mL/min/1.73 m^2^ would receive 4 points for the DM, 7.5 points for the HbA1c, 10 points for the TC, and 97.5 points for the eGFR (total = 119 points). The patient’s corresponding probability for developing CKD stages 3–5 at 5 years would be 65%.

## Discussion

In this adult outpatient cohort with preserved kidney function, the incidence of developing CKD stages 3–5 was approximately 1.4% per year. This finding was lower than the 2–4% per year that was reported in a recent systematic review that investigated CKD incidence among individuals with diabetes from 30 different countries [21]. The difference in the incidence was expected because the proportion of patients with diabetes was higher in the studies examined in the systematic review than in our cohort. Our study also demonstrated that among the traditional CVD risk factors, baseline eGFR, HbA1c, TC, and history of DM are strong predictors of CKD stages 3–5. Specifically, baseline eGFR is an important predictor of the development of these disease stages in both the general and high-risk population, such as patients with DM [21–25]. A decrease in GFR below a critical level results in a vicious cycle of worsening kidney function that contributes to HTN, which in turn perpetuates further nephron loss [2].

Several studies have shown that older age, DM, smoking, obesity, dyslipidemia, and HTN are independent risk factors for developing CKD stages 3–5 [22, 26–32]. From the age of 50 years, the lifetime risk of developing incident CKD is approximately 40% and could be even higher in the presence of additional risk factors, such as obesity, high blood pressure, or diabetes [33]. Interestingly, in our cohort of patients at risk of CVD, DM and baseline eGFR were the main predictors of CKD stages 3–5. Previous studies suggested that high-risk patients are more likely to die from CVD than develop kidney failure [7, 34, 35]; hence, survival bias may play a role in the lack of statistical significance of other risk factors in our study cohort. Nevertheless, the competing risk of death was accounted for in our study. Furthermore, DM has been shown to accelerate the progression of kidney function decline (2.1 and 2.7 mL/min/1.73 m^2^/year, respectively, for women and men with DM) [36] compared with essential HTN only (0.95 mL/min/year) [37] or with older age (0.75–1 mL/min/1.73 m^2^/year) [38]. Thus, factors that have a greater influence on kidney function may also increase the risk of premature death and therefore are important predictors of kidney failure. Our study also noted an inverse relationship between low cholesterol levels and incident CKD. This paradoxical finding could be explained by the confounding effects of malnutrition and chronic inflammation that are common in patients with CKD and end-stage renal disease [39].

Identification and risk stratification of CVD patients at risk of developing CKD stages 3–5 are important issues in clinical practice, particularly in outpatient clinics that provide care to patients with multiple vascular comorbidities. Furthermore, active detection of CKD risk and early treatment of risk factors may avoid complications associated with the subsequent CKD stages [33]. However, only a few studies have focused on predicting the risk of developing CKD stages 3–5 in patients at risk of CVD [40], and currently, the models developed to predict incident CKD require detailed laboratory and clinical information [30, 41]. In our study, we propose a simple and practical nomogram, which is based on four easily available clinical variables (i.e., HbA1c, DM, TC, and eGFR), to predict the incidence of CKD stages 3–5. This nomogram may help busy physicians triage high-risk patients toward more intensive testing and identify those who need early referral to a nephrologist.

One of the strengths of this study is that the diagnosis of CKD stages 3–5 was based on two consecutive readings of eGFR < 60 mL/min/1.73 m^2^, which were obtained ≥3 months apart. This could help account for intra-individual variability in eGFR and lead to a more accurate representation of kidney function. In addition, we used the CKD-EPI equation to define the outcome, which is more accurate than the Modification of Diet in Renal Disease Study equation according to most studies [11, 42–44]. Moreover, this study used documented anthropometric and laboratory measurements rather than self-reported information for both predictor variables and outcomes. Finally, standard receiver operating characteristic curve analysis assesses the predictive ability of a model within a fixed time horizon. In our study, we used time-dependent AUC analysis to assess the predictive accuracy of the nomogram at different time horizons.

This study has several limitations. First, other risk factors, such as albuminuria, were not explored. A number of studies have described the significance of albuminuria in predicting the development of kidney failure [30, 41, 45–47]; however, non-nephrologist physicians in the UAE reported that albuminuria is not routinely measured in their practice, and nearly 80% of physicians use eGFR alone as a screening tool for CKD [48]. Second, our sample size was modest compared to that in other studies. Finally, although the predictive power of the nomogram developed was thoroughly tested with internal validation, its applicability among a non-UAE patient population at risk of CVD remains to be investigated.

## Conclusions

This study demonstrated that in patients at high cardiovascular risk, eGFR, HbA1c, TC, and a history of DM are significant predictors of CKD stages 3–5. A simple and practical nomogram with good accuracy was constructed for predicting 5-year risk of developing CKD stages 3–5 based on competing risk model among patients with vascular comorbidities. This user-friendly risk prediction tool could help healthcare providers make better-informed decisions regarding CKD prevention and management in at-risk patients.

## Additional files

Additional file 1: Nomogram to predict the development of chronic kidney disease stages 3–5 at 5 years with a worked example. Instruction for use: locate a patient characteristic, such as history of diabetes, HbA1c, eGFR, and cholesterol levels, on the corresponding axis to determine the points the patient receives for each characteristic. Add the points of each characteristic and locate the sum on the total points axis. Draw a line straight down to identify the patient’s probability of developing CKD stages 3–5 at 5 years. *HbA1c* glycosylated hemoglobin A1c, *eGFR* estimated glomerular filtration rate. (TIF 60 kb)

Additional file 2: Use of estimated glomerular filtration rate to predict incident chronic kidney disease in patients at risk of cardiovascular disease: a retrospective study dataset. *ID* identification, *DM* diabetes mellitus, *HbA1c* glycosylated hemoglobin A1C, *CVD* cardiovascular disease, *HTN* hypertension, *SBP* systolic blood pressure, *DBP* diastolic blood pressure, *BMI* body mass index, *eGFR* estimated glomerular filtration rate, *CKD* chronic kidney disease. (XLSX 54 kb)

## Data Availability

The dataset supporting the conclusions of this article is included within the article and its Additional file 2.

## Abbreviations

AUC: Area under the curve
BMI: Body mass index
CI: Confidence interval
CKD: Chronic kidney disease;
CKD-EPI: CKD Epidemiology Collaboration
CVD: Cardiovascular disease
DBP: Diastolic blood pressure
DM: Diabetes mellitus
eGFR: Estimated glomerular filtration rate
EMR: Electronic medical record
HbA1c: Glycosylated hemoglobin A1C
HTN: Hypertension
SBP: Systolic blood pressure
SCr: Serum creatinine
SHR: sub-distribution hazard ratio
TC: Total cholesterol
TG: Triglycerides
UAE: United Arab Emirates
