# Supplementary figures and images for: Use of estimated glomerular filtration rate to predict incident chronic kidney disease in patients at risk of cardiovascular disease: a retrospective study
Source: BMC Nephrol. 2019 Aug 20;20:325. doi: 10.1186/s12882-019-1494-8 (PMC6700777; doi:10.1186/s12882-019-1494-8)

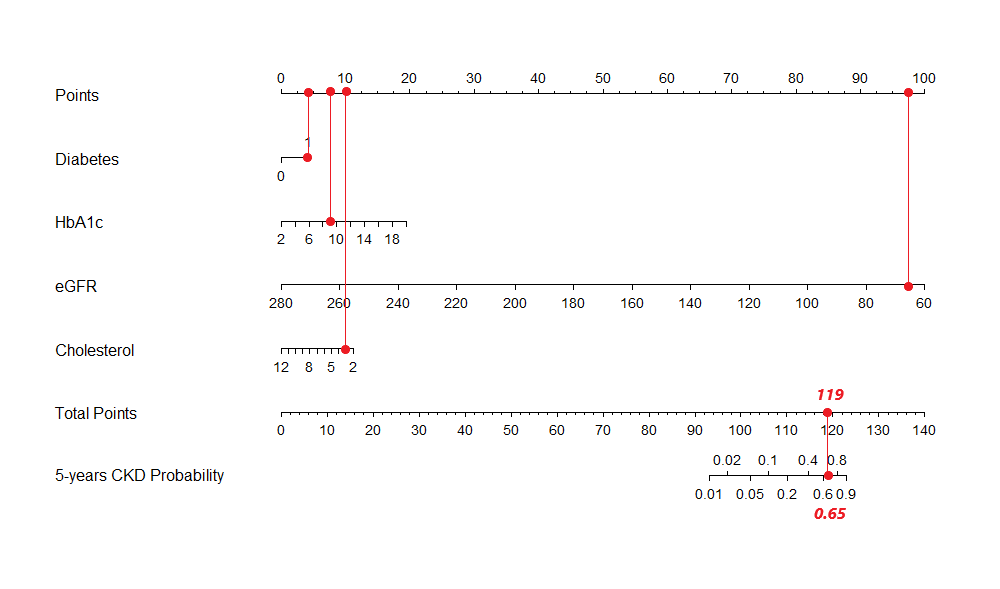

Supplement: Supplementary file 1 — Nomogram to predict the development of chronic kidney disease stages 3–5 at 5 years with a worked example. Instruction for use: locate a patient characteristic, such as history of diabetes, HbA1c, eGFR, and cholesterol levels, on the corresponding axis to determine the points the patient receives for each characteristic. Add the points of each characteristic and locate the sum on the total points axis. Draw a line straight down to identify the patient’s probability of developing CKD stages 3–5 at 5 years. HbA1c glycosylated hemoglobin A1c, eGFR estimated glomerular filtration rate. (TIF 60 kb) [file 12882_2019_1494_MOESM1_ESM.tif]
